# How effective are digital interventions in increasing flu vaccination among pregnant women? A systematic review and meta-analysis

**DOI:** 10.1093/pubmed/fdab220

**Published:** 2021-06-23

**Authors:** Jo Parsons, Sarah E Griffiths, Nicky Thomas, Helen Atherton

**Affiliations:** The Unit of Academic Primary Care, Warwick Medical School, The University of Warwick, Coventry CV4 7AL, UK; Leicester School of Nursing and Midwifery, De Montfort University, Leicester LE1 9BH, UK; Division of Life Sciences, The University of Warwick, Coventry, CV4 7AL, UK; The Unit of Academic Primary Care, Warwick Medical School, The University of Warwick, Coventry CV4 7AL, UK

**Keywords:** digital interventions, flu vaccination, pregnancy, systematic review

## Abstract

**Background:**

Flu can have serious consequences for pregnant woman and unborn babies. Vaccination provides safe and effective protection, yet uptake among pregnant women is below national targets. Digital interventions are effective at increasing adherence to health interventions.

**Aims:**

This review aimed to establish whether digital interventions are effective at increasing rates of flu vaccination among pregnant women, and to determine the overall effect size.

**Method:**

Systematic searches identified digital intervention trials, aiming to increase rate of flu vaccination among pregnant women. Random-effects meta-analysis provided a combined effect size and examined which mode of digital interventions had the largest effects on flu vaccination.

**Results:**

Ten studies were included in the review. The majority of digital interventions were more effective at increasing rates of flu vaccination (7–81.3% uptake) than usual care or non-digital interventions (7.3–47.1% uptake). When meta-analysed, digital interventions had a small, non-significant effect (odds ratio [OR] = 1.29, 95% confidence interval [CI]: 0.71, 2.31), *P* = 0.40. Text messages (OR = 1.25, 95% CI: 0.58, 2.67), *P* = 0.57 appeared less effective than other digital interventions (OR = 1.58, 95% CI: 1.02, 2.46), *P* = 0.04.

**Conclusions:**

Overall, there is a lack of high-quality studies reporting the effectiveness of digital interventions at increasing flu vaccination during pregnancy. Future interventions may benefit from using video or social media to communicate messages for maximum success in targeting an increase in rates of flu vaccination in pregnancy.

## Introduction

Pregnant women and their unborn babies are at increased risk of complications from flu, due to physiological and immunological changes occurring during pregnancy. Pregnant women are approximately four times more likely to be hospitalized with flu than non-pregnant women, and risk of death from flu is higher among pregnant women.^1–3^ Furthermore, there is an increased risk of premature birth, stillbirth and low birthweight for unborn babies, resulting from maternal flu.^1^ The flu vaccination has been shown to be safe and effective,^4–6^ yet uptake among pregnant women in England is annually below the 75% national target, with only 45.2% of pregnant women receiving the vaccination in 2018/19.^7^ Pregnant women with lower education, living at or below the poverty line, non-Hispanic or black ethnicity^8–10^ and smokers^11^ are less likely to have the flu vaccination during pregnancy. Pregnant women have been shown to underestimate their susceptibility to and the seriousness of flu while pregnant, which may influence their vaccination decisions.^12^^,^^13^

Internet use has increased rapidly over recent years, with 96% of households in Great Britain having internet access in 2020, compared to 56% in 2006.^14^ The popularity of internet use is expected to further increase, with nearly 54 million people estimated to use internet-enabled smartphones in the UK by 2022,^15^ making digital health interventions accessible to many people.

Digital interventions have proven effectiveness in increasing health behaviours such as smoking cessation,^16^ physical activity,^17^ physical activity in cardiovascular disease^18^ and asthma self-management.^19^ If digital interventions are an effective approach to increase flu vaccination among pregnant women, it suggests an accessible mechanism for primary care services to improve health of pregnant women and unborn babies, in turn reducing associated healthcare costs resulting from maternal flu. To date, effectiveness of digital interventions for increasing flu vaccination rates among pregnant women is yet to be determined.

This review aimed to establish whether digital interventions are effective at increasing flu vaccination rates among pregnant women, and to determine the size of the effect.

Review objectives:

(i) To examine the effectiveness of digital interventions for increasing rate of flu vaccination among pregnant women.
(ii) To compare the effectiveness of different types of digital interventions for increasing rate of flu vaccination among pregnant women.

## Method

This study was conducted in line with a pre-defined protocol^20^ and is reported in line with PRISMA guidelines.^21^

### Eligibility criteria

Studies testing effectiveness of digital interventions for increasing flu vaccination rate among pregnant women were eligible for inclusion. For the purposes of this review, the term ‘digital intervention’ is defined as an intervention that attempts to change pregnant women’s vaccination behaviour, delivered via digital or mobile devices directly to participants. This includes text messages (including text, video or audio-based messages), internet-delivered interventions (including websites, mobile applications (apps) or social media sites) and other digital strategies.^22^

Any comparison group was acceptable, including usual care, wait-list comparators, historical control groups (without digital intervention), digital interventions unrelated to flu vaccination or non-digital interventions. Only original research studies were eligible for inclusion, with systematic reviews, protocols, commentaries and conference abstracts excluded.

Studies were required to be randomized or non-randomized controlled trials, quasi-randomized controlled trials or other quantitative designs reporting rate of flu vaccination (e.g. before and after trials) following implementation of a digital intervention, which also contained a comparator. Case series and case reports were excluded. No date or country restrictions were included, but studies were required to be published in English. Full inclusion and exclusion criteria can be found in Table 1.

**Table 1:** Inclusion and exclusion criteria

*Inclusion criteria*	*Exclusion criteria*
Participants are pregnant women	Any participants other than pregnant women
Studies testing the effectiveness of a digital intervention to increase the rate of flu vaccination (if multiple types of intervention are tested, at least one of these needs to be a digital intervention, and results must allow for the rate of digital interventions to be extracted). Appropriate statistical information about the effectiveness is provided	No intervention is tested, none of the tested interventions are digital
Studies comparing the effectiveness of a digital intervention (e.g. text message, website, mobile app) to usual care, to a non-digital intervention or to a historical control group without digital intervention	No comparator, control or usual care condition is included
Outcome being studied is the rate of flu vaccination (either actual vaccination behaviour or intention to vaccinate)	The rate of flu vaccination is not the outcome measure
Original research studies only	Systematic reviews, protocols, commentaries, conference abstracts
Studies will be RCTs, non-RCTs, quasi-RCTs or other quantitative study	Other study designs (including quantitative studies that report audits, surveys and similar, or those that do not report the rate of flu vaccination after the implementation of a digital intervention

### Outcome measures

The primary outcome was rate of flu vaccination among pregnant women after receiving targeted digital interventions, compared to a comparator group. This could be either self-reported vaccination status or status obtained from electronic patient records. The secondary outcome was the size of the effect of digital interventions (using odds ratio [OR]).

### Information sources

The following electronic bibliography databases were searched: MEDLINE, Embase, Web of Science, Scopus, Cochrane database, PsycINFO and Cochrane Central Register of Controlled Trials (CENTRAL). In-progress trials were searched for on the clinical trials register. Searches were conducted in April 2020.

### Search strategy

Search terms included all possible terms relating to ‘vaccination’, ‘influenza’, ‘pregnancy’ and variations of ‘digital interventions’ to include interventions containing significant influence from text messages, video, Internet, or mobile phone apps.^22^^,^^23^ Reference sections of studies meeting inclusion criteria and papers citing studies meeting inclusion criteria were screened to identify other eligible studies. The full search strategy can be found in Supplemental 1.

### Data management and screening process

Results from database searches were combined and duplicates removed. Endnote X9 and Covidence software were used to organize data. Titles and abstracts of all search results were first screened to assess eligibility for inclusion in the review. Any studies that appeared to be eligible were subjected to the next stage of screening. Full text of studies were then obtained and screened against the predefined inclusion criteria. Screening was conducted by two researchers independently, and discrepancies were discussed until consensus was reached. This resulted in a full and final set of studies for inclusion in the review.

Data were then extracted from included studies. This step was conducted by two researchers independently, using a predefined extraction form. The following information was extracted from each study: author, publication year, study design, study setting, participants, intervention details (mode of digital intervention such as text message, video, mobile phone app), comparison/control condition, rate of flu vaccination and size of intervention effect (where reported). Any discrepancies in data extraction were discussed until a consensus was reached. Eligibility for inclusion in the meta-analysis was also determined for each study.

### Quality assessment

For randomized controlled trials, risk of bias was assessed using the Cochrane Risk of Bias Tool.^24^ Each study was rated as low, medium or high risk of bias on each domain. For non-randomized controlled trials, risk of bias was assessed using the Cochrane Risk of Bias in Non-Randomised Studies of Interventions.^25^ Each study was rated as low, moderate, serious or critical risk of bias, or categorized as no information to make a judgement for each domain.

Overall ratings of risk of bias were calculated by totalling numbers of domains for each paper rated as low risk, some concerns (or moderate risk) or high risk. Randomized controlled trials were deemed to be as follows: ‘low risk’, if all domains were rated low risk; ‘some concerns’, if at least one domain was rated some concerns but no domains rated high risk and ‘high risk’, if at least one domain was rated high risk or multiple domains were rated as some concerns.^24^ Non-randomized controlled trials were deemed to be as follows: ‘low risk’, if all domains were rated low risk; ‘moderate risk’, if all domains are rated low or moderate risks; ‘serious risk’, if there was at least one domain rated serious risk of bias but no ratings of critical risk and ‘critical risk’, if at least one domain was rated critical risk.^25^ Quality was assessed by two authors independently. Any discrepancies were discussed until consensus was reached.

### Data synthesis

Key information extracted from included studies was synthesized, including descriptive information about type and content of intervention and control conditions for each study. Rates of flu vaccination were extracted and synthesized to determine the effectiveness of digital interventions at increasing flu vaccination among pregnant women. Summaries of risk of bias of included studies were reported.

### Data analysis

Heterogeneity was assessed using meta-analysis software.

RevMan software version 5.4.1 was used to calculate OR for each digital intervention, using a random-effects model. Where studies included more than one digital intervention, the most digitally intensive intervention was included in the meta-analysis.

A sensitivity analysis was conducted to examine whether risk of bias of included studies affected the overall effect size of digital interventions on flu vaccination rate. A moderator analysis was conducted to examine differences in effects between types of digital interventions and to determine which mode of delivery is more effective in increasing the rate of flu vaccination among pregnant women.

## Results

### Main characteristics of included studies

A total of 479 results (after duplicates were removed) were subjected to title and abstract screening. Of these, 33 full-text papers were obtained and screened against the eligibility criteria. Ten studies met all inclusion criteria and were included in the review. The number and reasons for exclusion can be seen in the PRISMA flowchart found in Supplemental 2. 'Reasons for exclusions include the study having the wrong population, i.e. not pregnant women, or a study design not meeting the inclusion criteria for the review.

Of the 10 included papers, 9 consisted of randomized controlled trials,^26–34^ while 1 was a non-randomized trial (quantitative retrospective study).^35^ Eight of the included studies were conducted in the USA,^26–31^^,^^33^^,^^35^ one in Australia^32^ and one in Canada.^34^ Eight of the studies were conducted from 2015 onwards,^26–29^^,^^31^^,^^32^^,^^34^^,^^35^ while two were conducted before 2015.^30^^,^^33^ There were 9831 participants across the 10 included studies. Seven of the studies had less than 350 participants, while the remaining three studies^29^^,^^33^^,^^35^ had between 1000 and 4000 per study. Five studies reported a mean age of participants,^26–28^^,^^31^^,^^34^ ranging from 26 to 32 years.

The majority of included studies were set in hospital or clinic settings,^26–28^^,^^30–34^ while one involved current enrolees of the Text4baby Service (a free national mobile health service in the USA),^29^ and one involved a national internet survey.^35^ Five studies employed objective measures of vaccination uptake, verified by patient records or monitoring uptake on the day of the study,^28^^,^^30–33^ and four used self-reported measures.^27^^,^^29^^,^^34^^,^^35^ One study used three methods of obtaining rate of vaccination (self-report, reviewing of electronic records and verification via a local vaccination register).^26^ Full characteristics of studies can be found in Table 2.

**Table 2:** Table of characteristics

*Study author and year*	*Study design*	*Country of study*	*Study setting*	*Participants*	*Intervention condition (including mode of digital intervention)*	*How/when delivered*	*Control/comparator condition*	*Type of measure (self-report or objective)*
Bushar 2017	Non RCT (Quantitative retrospective study)	USA (nationwide)	National survey data. Internet panel surveys conducted by CDC	Pregnant women 18–49 years. Text4baby recallers *n* = 377 Non-participants *n* = 2,824	Text4baby messages. Vaccination encouragement messages. Tailored education and opportunity to schedule vaccination	Text messages received at home during current/most recent pregnancy	Women not receiving messages or not participating in text4baby	Self-report
Chamberlain 2015	Cluster RCT	USA (Georgia)	Obstetric practices	Mean age of participants = 27.2. Total participants: 325 Intervention = 161 Control = 164	iBook-based app. Patient-level: iPad-based interactive tool (text and audio covering the importance of vaccination in pregnancy, dangers of flu, safety of vaccination, testimonials and video of obstetrician), maps to local pharmacies/health departments providing vaccines Practice-level: vaccine champion, lapel buttons, posters, brochures Provider-level: provider-to-patient talking points, peer-to-peer promotion education	Study conditions. iPads distributed to pregnant women in examination rooms while waiting to be seen by a physician	Usual care: Control group practices did not receive any package materials for the duration of the study. They were requested to maintain their standard of care regarding influenza and/or Tdap vaccine promotion and administration	Both: Vaccine receipt was assessed in 3 ways: obstetric chart review if the vaccine(s) were stocked by the patient’s obstetric practice, patient recall during a follow-up survey conducted 2–3 months post-partum and queries to the Georgia Registry for Immunization Transactions and Services
Frew 2016	RCT	USA (Atlanta, Georgia)	Participants recruited from antenatal practices located in urban and suburban areas	Black/African American pregnant women. Mean age = 26.1 95 randomly assigned: 31 to affective messaging intervention, 30 to cognitive messaging intervention, 34 to comparison	Videos. Affective messaging: persuasive scenario information (using cognitive dissonance, Cueing techniques, normative beliefs, etc.) Cognitive messaging: Short factual Q&A with physicians covering safety and recommendation information. Promoting issue-relevant thinking	Study conditions: Participants watched video on study iPad in study waiting room	Paper-based materials, consisting of generic influenza information developed by CDC: ‘2012/13 influenza VIS’ (Vaccine Information Statements): Those assigned to the VIS arm were given the material to read in the presence of a study team member	Self-report
Goodman 2015	RCT	USA (Cleveland)	Three suburban antenatal clinics (including 11 eligible providers).	Mean age = 31. 105 participants were randomized; 53 to intervention condition, 52 to control condition.	Video. Educational video developed by CDC ‘Protect yourself, protect your baby’, 3½ minutes, addressing vaccination health beliefs concepts found to be predictive of vaccination	Study conditions: Those potentially interested were escorted into a designated study room	Video. ‘Put your hands together’, CDC video of the same length addressing hand washing hygiene	Objective/verified vaccination on day of study
Jordan 2015	RCT	USA (nationwide)	Pregnant women currently enrolees to the Text4baby service	Mean age of participants not reported. Planning to vax: Usual message: 1,360 Enhanced message: 292 Not planning to vax: Usual message: 1,228 Tailored message: 1,025. Total participants = 3,905.	Text message. Those intended to get flu vaccination assigned to encouragement message or encouragement message plus opportunity to schedule a reminder at follow-up. Those not intending to have flu vaccination assigned to either general educational message or educational message tailored to their reason for non-vaccination (if they provided one).	Remote messages sent to Text4baby users (not study conditions)	Text message. Usual care messages. Planning to vaccinate; received one encouragement message to put a reminder on their calendar. Not planning to vaccinate; received general message stressing importance of influenza vax.	Self-report
Moniz 2013	RCT	USA (Pittsburgh)	Routine obstetric visits to one women’s hospital outpatient clinic	Women between the ages of 13 and 49 years, who were pregnant at less than 28 weeks gestation. 204 ITT (100 to general—control, 104 to flu—intervention)	Text messages. 12 × weekly messages regarding general preventive health in pregnancy plus importance of influenza vaccination in pregnancy. General preventive health messages received by all participants included importance of prenatal vitamins, nutritional foods, and seat belt use during pregnancy. The Flu group received additional information in each weekly text message addressing the benefits and safety of influenza vaccination during pregnancy	Remote messages sent weekly	Text messages. Text messages regarding general preventive health in pregnancy. The general preventive health messages received by all participants covered topics such as the importance of prenatal vitamins, nutritional foods and seat belt use during pregnancy	Objective measure.
O’Leary 2019	RCT	USA (Colorado)	Pregnant women from integrated healthcare system in Colorado	Mean age = 32. Women in the third trimester of pregnancy. Total *n* = 289. Vaccine social media *n* = 140, Vaccine information. *n* = 105, usual care *n* = 44.	Website and social media. Arm 1: Website with vaccine information and social media components (VSM). Also had access to interactive components including blog discussion forum, chat room and ‘ask a question’ portal Arm 2: Website with vaccine information only Website also contained information specifically related to maternal vaccinations and concerns (national vaccine recommendations during pregnancy, safety info, ingredients, description of the disease and answers to common questions).	Remote. Links to intervention sent by email	Usual/routine obstetric care. All participants in usual care received routine obstetric care but did not have access to website intervention.	Objective ‘Vaccination data were extracted from the electronic health record’
Regan 2017	RCT	Australia (Western)	Nine practices in the Perth Metropolitan area and one rural practice	Pregnant women aged 18–44 years. Intervention condition *n* = 115, control condition *n* = 124. Total participants = 239	Text message. SMS message reminded patients of their eligibility for free influenza vaccine and prompted them to book an appointment	Remote delivery. One text message sent from patient’s medical practice	No text message.	Objective measure (Data extracted from medical records)
Stockwell 2014	RCT	USA (New York)	Obstetric patients from 5 community-based clinics in New York city.	Pregnant women aged < 20 to >40 who had a first trimester obstetric visit between 1 February and 31 August 2011. 1187 pregnant women randomized. 593 assigned to intervention group, 594 assigned to usual care group	Text message. 5 × automated message reminders. Due for vaccine, 3 × educational info messages, final message interactive with option to receive more info, side effects, etc. Unvaccinated women also received two message appointment reminders. Both groups received routine pre- and postnatal apt reminders	Remote/automated text messages	Usual care. Women in both groups received routine automated telephone pre- and postnatal appointment reminders provided directly from the clinic network	Objective measures from hospital immunization information system
Yudin 2017	RCT	Canada (Toronto)	Hospital-based antenatal clinic at a women’s health ambulatory care clinic in downtown Toronto serving a multi-ethnic patient population of varied socioeconomic status	Mean age of participants = 32. Pregnant women attending hospital-based antenatal clinic. Final analysis based on 281 participants, consisting of 129 in the text message group and 152 women in the control group.	Text message. Twice weekly messages for 4 weeks (max 8 messages). Specifically focused on influenza and vaccine. Messages emphasized the susceptibility of pregnant women to flu, effectiveness of the vaccine and poor outcomes in mother and baby, safety of the vaccine and that it is recommended for pregnant women	Text messages sent remotely	No intervention. No text message sent to participants in the control group	Self-report

### Digital interventions

The most common mode of digital intervention used in the included studies was text messages.^29^^,^^30^^,^^32–35^ Other methods of intervention included videos,^27^^,^^28^ website or social media^31^ and an iBook-based app.^26^ Interventions in three studies were delivered face-to-face in study conditions,^27^^,^^28^ and in examination rooms while waiting to be seen by a physician.^26^ The remaining seven studies involved interventions being delivered remotely, consisting of text messages or links being sent from the study team to participants at home.^29–35^

Comparators used in included studies included no intervention or usual care,^26^^,^^29^^,^^31–35^ non-digital interventions^27^ and non-vaccination-related interventions.^28^^,^^30^ Details of intervention and comparator conditions are available in Table 2.

### Quality assessment

Five studies were given an overall rating of high risk of bias.^26^^,^^27^^,^^29^^,^^31^^,^^34^ Three were given an overall rating of some concerns or moderate risk of bias,^32^^,^^33^^,^^35^ and two studies were given an overall rating of low risk of bias.^28^^,^^30^ The domain with the most occurrences of potential bias was ‘Risk of bias arising from the randomisation process’.^24^ Individual domain ratings and overall risk of bias ratings for each study can be seen in Table 3.

**Table 3:** Risk of bias ratings

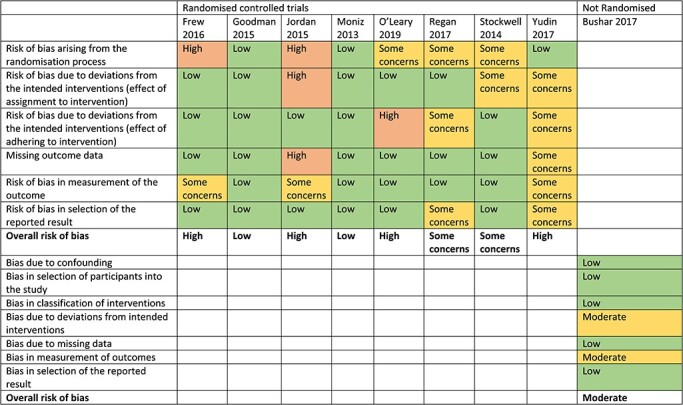

### Effectiveness of digital interventions

The rate of vaccination (reported as percentage of pregnant women within the sample receiving the flu vaccination) in included studies ranged between 7% (reported by the iBook condition in Frew *et al*.’s study)^27^ and 81.3%^35^ in intervention conditions and between 7.3^26^ and 47.1%^35^ in control conditions. Full flu vaccination uptake rates can be found in Table 4. Overall rates of vaccination suggest that the majority of intervention conditions were more effective than control conditions^26–28^^,^^31^^,^^33–35^ at increasing flu vaccination uptake among pregnant women. This shows that digital interventions are often a more effective approach than non-digital or no intervention.

**Table 4:** Rate of vaccination uptake

*Study*	*Rate of flu vaccination*	*Size of intervention effect*
Bushar 2017	Influenza vaccination coverage for Text4baby recallers was 81.3% compared with 47.1% for non-participants. Received recommendation and offer: Text4baby influenza message recaller: 314 Text4baby non-participant: 1,551 Received recommendation but no offer: Text4baby influenza message recaller: 31 Text4baby non-participant: 462 No recommendation or offer: Text4baby influenza message recaller: 27 Text4baby non-participant: 728	Text4baby recallers AND offer were more likely to report influenza vaccination than non-participants (adjusted prevalence ratios (APR) = 1.29, 95% CI = 1.21, 1.37). Among those that received provider recommendation but no offer to vaccinate, Text4baby recallers were more likely to report vaccination (APR = 1.52, 95% CI = 1.07, 2.17). Among those who received neither provider recommendation or offer to vaccinate, Text4baby recallers were more than three times as likely to report receipt of vaccination (APR = 3.39, 95% CI = 2.03, 5.67)
Chamberlain 2015	Intervention 16/149 10.7%, Control 11/151 7.3% More intervention group women received antenatal influenza and Tdap vaccines than did control group women, but the absolute risk difference before and after adjustment for the clustered study design were small and non-significant	Study-adjusted antenatal influenza RD: 3.6%, 95% CI: −4.0, 11.2
Frew 2016	Influenza vaccine administered during pregnancy: Comparison group: *n* = 4, 12% Pregnant pause movie: *n* = 4, 13% Vaccines for a healthy pregnancy iBook: *n* = 2, 7%	Arm 2: Risk ratios (RR): 1.10 (95% CI = 0.30, 4.01); Arm 3: RR 0.57 (95% CI = 0.11, 2.88) Arm 1 (control): 4/34 (12%); Arm 2: 4/31 (13%); Arm 3: 2/30 (7%)
Goodman 2015	Intervention condition: 28% (15/53) participants had the flu vaccination during the office visit. Control condition: 25% (13/52) participants had the flu vaccination during the office visit	Not reported
Jordan 2015	For both planning and not planning: Usual message: 1088/2588 (42.0%) Enhanced: 390/1317 (29.6%) General: 125 (59%) Specific: 46 (57%)	Among pregnant women, receipt of any enhanced reminder increased the odds of influenza vaccination at follow-up (adjusted odds ration (AOR) = 1.5, CI = 1.4, 3.1) Pregnant women who received a general reminder had higher odds of continual intent (AOR = 1.8, 95% CI = 1.1, 2.9) Pregnant women who received a specific reminder had increased offs of continued intent (AOR = 3.1, 95% CI = 1.4, 6.8) Also had higher odds of continued intent than actual vaccination (AOR = 2.0, 95% CI = 1.1, 3.3).
Moniz 2013	General: Non-vaccinated *n* = 69, 69% Vaccinated *n* = 31, 31% Flu: Non-vaccinated *n* = 70, 67% Vaccinated *n* = 34, 33% The overall influenza vaccination rate among participants was 32% with no difference between participants in the General (31% [*n* = 31]) compared with Flu (33% [*n* = 34]) groups (difference 1.7%, 95% CI 211.1–14.5%)	Not reported
O’Leary 2019	54% overall received the influenza vaccine before delivery (155/289). Both intervention arms achieved higher vaccination than usual care. VSM arm: 57% (80/140), VI arm: 56% (59/105) and usual care: 36% (16/44)	Odds of influenza vax were twice as high in the VSM arm that the UC are (OR = 2.19, 95% CI = 1.06, 4.53) and the VI versus the UC arm (OR = 2.20, 95% CI = 1.03, 4.69). There was no difference between the intervention arms
Regan 2017	Intervention group: total *n* = 115, vaccinated n = 20 (17.4%) Control group: total *n* = 124, vaccinated n = 24 (19.3%)	Absolute risk difference: −2.7% RR = 0.90, 95% CI = 0.53, 1.54
Stockwell 2014	(December 31): Intervention: Total *n* = 576. Vaccinated *n* = 284 (49.3%). Usual Care: Total *n* = 577. Vaccinated *n* = 269 (46.6%).	Relative risk = 1.06, 95% CI = 0.94, 1.19) Other rates available in Table 2
Yudin 2017	Overall influenza vaccination rate in the whole sample was 29%, with no significant difference in rates between intervention group: 40/129 = 31% and control group: 41/152 = 27%.	Not reported

### Meta-analysis

All 10 studies were included in the meta-analysis. Digital interventions had a small, non-significant effect on flu vaccination among pregnant women (OR = 1.29, 95% CI: 0.71, 2.31), *P* = 0.40, *I*^2^ = 96%. (see Supplemental 3 for meta-analysis forest plot).

### Additional analyses

A sensitivity analysis examined whether the effect of digital interventions was increased when studies rated as high risk of bias were removed from the meta-analysis. Removing the five high risk of bias studies resulted in a larger effect of digital interventions on the rate of flu vaccination. However, this effect was still non-significant (OR = 1.47, 95% CI: 0.65, 3.34), *P* = 0.35, *I*^2^ = 95%. See Supplemental 4 for sensitivity analysis forest plot.

A moderator analysis was conducted to examine whether there was a difference in effectiveness depending on the type of digital intervention used. Six studies used text message-based interventions, and these had a smaller, non-significant effect on flu vaccination uptake (OR = 1.25, 95% CI: 0.58, 2.67), *P* = 0.57, *I*^2^ = 97%, than all other modes (video, social media and iBook) of digital interventions (OR = 1.58, 95% CI: 1.02, 2.46), *P* = 0.04, *I*^2^ = 2%. See Supplementa l 5 for moderator analysis forest plots.

### Heterogeneity

A very high level of heterogeneity (*I*^2^ > 75%^36^) was present in the effect of digital interventions for flu vaccination (*I*^2^ = 96%). As heterogeneity was above 75%, a random-effects model was used.

### Publication bias

Examination of the funnel plot (see Supplemental 6) suggests the presence of asymmetry across studies, possibly indicating some publication bias, and some missing unpublished studies with negative effects. The analysis of funnel plots however can be subjective and difficult to interpret.^37^

## Discussion

### Main findings of the study

The majority of individual digital interventions were more effective at increasing flu vaccination among pregnant women than usual care or non-digital interventions. However, when the studies were pooled and weighted in the meta-analysis, there was a small non-significant effect. There was considerable heterogeneity in the results (particularly in those using text message interventions), and these findings are likely to be attributable to the small sample sizes found in more than half of the included studies and differences in interventions. This highlights the need for further, well-conducted studies with larger sample sizes.

A moderator analysis examining the effectiveness of different types of digital interventions showed that text messages were less effective than other modes of intervention, although there was significant heterogeneity present. This is particularly interesting as more than half of the digital interventions in this study used text messages to convey the digital message; the use of text messages is generally a popular approach for digital health interventions, yet in this review they were less effective than videos, social media and iBooks. This finding differs to findings of previous meta-analyses, which found that text message-based interventions were more effective at changing health behaviours than other modes of digital interventions.^16^^,^^38^

### What is already known on this topic

The susceptibility of pregnant women to flu and the effectiveness of digital interventions for some health behaviours are well known, yet little is known about the effectiveness of digital interventions in increasing flu vaccination uptake among this population.

Differences between findings of the current review and previous reviews in the effectiveness of text message interventions in changing behaviour may be explained by the type of behaviour being examined. Previous research has suggested that health-related interventions conveying risk are more effective when engaging and visual information is used.^39^^,^^40^ This may explain why visual interventions (such as video, social media and iBooks) for flu vaccination are more effective than text messages, which are limited to the presentation of facts and statistics. Visual and engaging interventions are not easily communicated using text messages alone.

### What this study adds

This study increases knowledge around appropriate approaches to increase flu vaccination among this population, potentially influencing clinical practice and service improvement for this under-researched area. This can ultimately have a positive impact on the rate of flu vaccination uptake, improving health and reducing mortality of pregnant women and unborn babies.

The majority of studies included in this review showed that digital interventions were more effective at increasing the rate of flu vaccination, when compared to non-digital interventions or usual care. This suggests that campaigns and interventions aiming to increase flu vaccination for this population may benefit from including digital components: specifically, videos, social media and iBooks, rather than text messages. This has practical implications for recommended content of new interventions in development, both for routine vaccinations during pregnancy and for the development of interventions for new diseases, such as for the new COVID-19 vaccination.

Although the majority of included studies showed that digital interventions were more effective at increasing flu vaccination among pregnant women, when the studies were pooled and weighted for the meta-analysis, there was no effect compared to non-digital interventions or usual care. This contradicts previous research showing digital interventions improve health-related behaviours.^16–19^ This may be due in part to the small number of included studies, highlighting the need for more research examining the effectiveness of digital interventions for flu vaccination in pregnancy.

### Limitations of this study

Many of the studies included in this review have small sample sizes, which may contribute to the non-significant effect of digital interventions in increasing flu vaccination in this study. There are likely to be differences between studies that provide interventions in study or clinical settings compared to those delivered remotely. The presence of experimental settings or researchers may impact uptake of vaccination.

Comparators or level of usual care also varied significantly between studies. Some involved no information or general health information, whereas others provided information about flu, which may have more impact on intention to vaccinate. The majority of studies were conducted in the USA. There is the potential that this country has different levels of usual care or better access to Internet than other countries. More research is needed in other countries to see if digital interventions are effective there (e.g. in remote populations where usual care may be considerably more limited). Additional research in the UK would be beneficial to support NHS maternity care for mothers and babies, as this is currently lacking.

## Conclusion

While digital interventions had proven efficacy for some health behaviours, effectiveness over other interventions for increasing flu vaccination in pregnancy had not previously been established. This review showed that digital interventions taken individually were generally more effective at increasing flu vaccinations among pregnant women, but the overall pooled and weighted effect was small and non-significant. Text messages appeared to be less effective than other digital methods at increasing flu vaccination among this population, providing valuable insight for future digital interventions.

## Supplementary Material

Supplemental_1_search_strategy_fdab220

Supplemental_2_flowchart_fdab220

Supplemental_3_forest_plots_fdab220

Supplemental_4_sensitivity_forest_plot_fdab220

Supplemental_5_moderator_analysis_forest_plot_fdab220

Supplemental_6_funnel_plot_fdab220

Supplemental_7_PRISMA_checklist_fdab220

## Notes

**Jo Parsons**, Research Fellow

**Sarah E. Griffiths**, Research Fellow

**Nicky Thomas**, PhD Student

**Helen Atherton**, Associate Professor of Primary Care Research
