# Supplementary material for: How effective are digital interventions in increasing flu vaccination among pregnant women? A systematic review and meta-analysis
Source: J Public Health (Oxf). 2021 Jun 23;44(4):863–76. doi: 10.1093/pubmed/fdab220 (PMC9715302; doi:10.1093/pubmed/fdab220)
Supplement: Supplemental_1_search_strategy_fdab220 [file supplemental_1_search_strategy_fdab220.docx]

Supplemental 1: Search strategy

Searches run: 08 April 2020

|  | Number of references | De-duplicated references |
| --- | --- | --- |
| Medline | 91 | 90 |
| PreMedline | 23 | 20 |
| Embase | 301 | 217 |
| CINAHL | 193 | 119 |
| Web of Science | 117 | 36 |
|  |  |  |
| TOTAL | 725 | 482 |

Database: Ovid MEDLINE(R) <1946 to March Week 4 2020>

Search Strategy:

--------------------------------------------------------------------------------

1 exp Influenza Vaccines/ or flu.mp. or exp Influenza, Human/ or influenza.mp. (104544)

2 exp Pregnancy/ or pregnancy.mp. (927256)

3 pregnant women.mp. or exp Pregnant Women/ (83211)

4 pregnan*.mp. (942979)

5 exp prenatal care/ or antenatal.mp. or ante-natal.mp. or ante natal.mp. or prenatal.mp. or pre-natal.mp. or pre natal.mp. (178210)

6 exp maternal health/ or exp maternal welfare/ or maternal.mp. (277230)

7 2 or 3 or 4 or 5 or 6 (1064747)

8 1 and 7 (3658)

9 digital.mp. (105015)

10 apps.mp. (3394)

11 app.mp. (19703)

12 video*.mp. (140122)

13 (telemedicine or tele-medicine or tele medicine).mp. or exp Telemedicine/ (28949)

14 software.mp. or exp Software/ (242301)

15 computer*.mp. (758762)

16 exp Computers/ (77488)

17 web.mp. or exp Web Browser/ (86290)

18 (telehealth or tele health or tele-health).mp. (3499)

19 (text or texts or texting).mp. [mp=title, abstract, original title, name of substance word, subject heading word, floating sub-heading word, keyword heading word, organism supplementary concept word, protocol supplementary concept word, rare disease supplementary concept word, unique identifier, synonyms] (62785)

20 9 or 10 or 11 or 12 or 13 or 14 or 15 or 16 or 17 or 18 or 19 (1217190)

21 8 and 20 (91)

Database: Ovid MEDLINE(R) Daily Update <April 06, 2020>, Ovid MEDLINE(R) Epub Ahead of Print and In-Process & Other Non-Indexed Citations <April 06, 2020>

Search Strategy:

--------------------------------------------------------------------------------

1 exp Influenza Vaccines/ or flu.mp. or exp Influenza, Human/ or influenza.mp. (10766)

2 exp Pregnancy/ or pregnancy.mp. (40540)

3 pregnant women.mp. or exp Pregnant Women/ (11319)

4 pregnan*.mp. (50995)

5 exp prenatal care/ or antenatal.mp. or ante-natal.mp. or ante natal.mp. or prenatal.mp. or pre-natal.mp. or pre natal.mp. (14718)

6 exp maternal health/ or exp maternal welfare/ or maternal.mp. (29670)

7 2 or 3 or 4 or 5 or 6 (71782)

8 1 and 7 (400)

9 digital.mp. (26484)

10 apps.mp. (2331)

11 app.mp. (5548)

12 video*.mp. (24279)

13 (telemedicine or tele-medicine or tele medicine).mp. or exp Telemedicine/ (2596)

14 software.mp. or exp Software/ (35802)

15 computer*.mp. (43604)

16 exp Computers/ (41)

17 web.mp. or exp Web Browser/ (24347)

18 (telehealth or tele health or tele-health).mp. (1208)

19 (text or texts or texting).mp. [mp=title, abstract, original title, name of substance word, subject heading word, floating sub-heading word, keyword heading word, organism supplementary concept word, protocol supplementary concept word, rare disease supplementary concept word, unique identifier, synonyms] (26448)

20 9 or 10 or 11 or 12 or 13 or 14 or 15 or 16 or 17 or 18 or 19 (170482)

21 8 and 20 (23)

Database: Embase Classic+Embase <1947 to 2020 Week 14>

Search Strategy:

--------------------------------------------------------------------------------

1 influenza.mp. or exp influenza vaccine/ or exp influenza/ or exp influenza vaccination/ (162101)

2 flu.mp. or influenza/ (91667)

3 1 or 2 (180326)

4 pregnancy.mp. or exp pregnancy/ (990924)

5 pregnant wom?n.mp. or exp pregnant woman/ (157432)

6 pregnan*.mp. (1079519)

7 (prenatal or pre-natal or pre natal).mp. or exp prenatal care/ (299268)

8 (antenatal or ante-natal or ante natal).mp. (52189)

9 exp maternal care/ or exp maternal welfare/ or maternal.mp. or exp maternal morbidity/ (405053)

10 4 or 5 or 6 or 7 or 8 or 9 (1365195)

11 (telehealth or tele-health or tele health).mp. or exp telemedicine/ or exp telehealth/ (44556)

12 (telemedicine or tele-medicine or tele medicine).mp. (28483)

13 (app or apps).mp. [mp=title, abstract, heading word, drug trade name, original title, device manufacturer, drug manufacturer, device trade name, keyword, floating subheading word, candidate term word] (38764)

14 (text or texts or texting).mp. [mp=title, abstract, heading word, drug trade name, original title, device manufacturer, drug manufacturer, device trade name, keyword, floating subheading word, candidate term word] (100095)

15 digital.mp. (193853)

16 video*.mp. (199597)

17 exp software/ or software*.mp. (356545)

18 computer*.mp. or exp computer/ (1580842)

19 web.mp. or exp web browser/ (142657)

20 11 or 12 or 13 or 14 or 15 or 16 or 17 or 18 or 19 (2318681)

21 3 and 10 and 20 (301)

**TOPIC:** (flu or influenza) *AND* **TOPIC:** (pregnan* or antenatal or ante-natal or "ante natal" or prenatal or pre-natal or "pre natal" or maternal) *AND* **TOPIC:** (telemedicine or tele-medicine or "tele-medicine"or telehealth or tele-health or "tele health" or digital or computer* or software* or app or apps or text or texts or texting or video* or web or "web browser*")

CINAHL

| S1 | (MH "Influenza Vaccine") OR (MH "Influenza, Human") OR (MH "Influenza") OR "flu" | 25,166 | [Edit](javascript:__doPostBack('ctl00$ctl00$MainContentArea$MainContentArea$editControl$printHistory$HistoryRepeater$ctl00$linkEditSearch',''))S1 |
| --- | --- | --- | --- |
| S2 | "influenza" | 30,727 | [Edit](javascript:__doPostBack('ctl00$ctl00$MainContentArea$MainContentArea$editControl$printHistory$HistoryRepeater$ctl01$linkEditSearch',''))S2 |
| S3 | S1 OR S2 | 32,422 | [Edit](javascript:__doPostBack('ctl00$ctl00$MainContentArea$MainContentArea$editControl$printHistory$HistoryRepeater$ctl02$linkEditSearch',''))S3 |
| S4 | (MH "Pregnancy+") | 217,469 | [Edit](javascript:__doPostBack('ctl00$ctl00$MainContentArea$MainContentArea$editControl$printHistory$HistoryRepeater$ctl03$linkEditSearch',''))S4 |
| S5 | (MH "Expectant Mothers") | 8,073 | [Edit](javascript:__doPostBack('ctl00$ctl00$MainContentArea$MainContentArea$editControl$printHistory$HistoryRepeater$ctl04$linkEditSearch',''))S5 |
| S6 | "pregnan*" | 247,220 | [Edit](javascript:__doPostBack('ctl00$ctl00$MainContentArea$MainContentArea$editControl$printHistory$HistoryRepeater$ctl05$linkEditSearch',''))S6 |
| S7 | "maternal" | 105,049 | [Edit](javascript:__doPostBack('ctl00$ctl00$MainContentArea$MainContentArea$editControl$printHistory$HistoryRepeater$ctl06$linkEditSearch',''))S7 |
| S8 | (MH "Prenatal Care") | 17,429 | [Edit](javascript:__doPostBack('ctl00$ctl00$MainContentArea$MainContentArea$editControl$printHistory$HistoryRepeater$ctl07$linkEditSearch',''))S8 |
| S9 | pre-natal or prenatal or "pre natal" | 55,088 | [Edit](javascript:__doPostBack('ctl00$ctl00$MainContentArea$MainContentArea$editControl$printHistory$HistoryRepeater$ctl08$linkEditSearch',''))S9 |
| S10 | antenatal or ante-natal or "ante natal" | 15,048 | [Edit](javascript:__doPostBack('ctl00$ctl00$MainContentArea$MainContentArea$editControl$printHistory$HistoryRepeater$ctl09$linkEditSearch',''))S10 |
| S11 | S4 OR S5 OR S6 OR S7 OR S8 OR S9 OR S10 | 310,740 | [Edit](javascript:__doPostBack('ctl00$ctl00$MainContentArea$MainContentArea$editControl$printHistory$HistoryRepeater$ctl10$linkEditSearch',''))S11 |
| S12 | (MH "Telemedicine+") OR (MH "Telehealth+") | 23,573 | [Edit](javascript:__doPostBack('ctl00$ctl00$MainContentArea$MainContentArea$editControl$printHistory$HistoryRepeater$ctl11$linkEditSearch',''))S12 |
| S13 | telemdicine or tele-medicine or "tele medicine" or telehealth or tele-health or "tele health" | 17,927 | [Edit](javascript:__doPostBack('ctl00$ctl00$MainContentArea$MainContentArea$editControl$printHistory$HistoryRepeater$ctl12$linkEditSearch',''))S13 |
| S14 | (MH "Mobile Applications") | 7,083 | [Edit](javascript:__doPostBack('ctl00$ctl00$MainContentArea$MainContentArea$editControl$printHistory$HistoryRepeater$ctl13$linkEditSearch',''))S14 |
| S15 | "app or apps" | 11 | [Edit](javascript:__doPostBack('ctl00$ctl00$MainContentArea$MainContentArea$editControl$printHistory$HistoryRepeater$ctl14$linkEditSearch',''))S15 |
| S16 | "computer*" | 168,898 | [Edit](javascript:__doPostBack('ctl00$ctl00$MainContentArea$MainContentArea$editControl$printHistory$HistoryRepeater$ctl15$linkEditSearch',''))S16 |
| S17 | (MH "Software+") OR "software*" | 505,837 | [Edit](javascript:__doPostBack('ctl00$ctl00$MainContentArea$MainContentArea$editControl$printHistory$HistoryRepeater$ctl16$linkEditSearch',''))S17 |
| S18 | (MH "Videorecording+") OR "video*" | 69,376 | [Edit](javascript:__doPostBack('ctl00$ctl00$MainContentArea$MainContentArea$editControl$printHistory$HistoryRepeater$ctl17$linkEditSearch',''))S18 |
| S19 | (MH "Text Messaging+") | 3,004 | [Edit](javascript:__doPostBack('ctl00$ctl00$MainContentArea$MainContentArea$editControl$printHistory$HistoryRepeater$ctl18$linkEditSearch',''))S19 |
| S20 | "text or texts or texting" | 0 | [Edit](javascript:__doPostBack('ctl00$ctl00$MainContentArea$MainContentArea$editControl$printHistory$HistoryRepeater$ctl19$linkEditSearch',''))S20 |
| S21 | "digital" | 41,666 | [Edit](javascript:__doPostBack('ctl00$ctl00$MainContentArea$MainContentArea$editControl$printHistory$HistoryRepeater$ctl20$linkEditSearch',''))S21 |
| S22 | (MH "World Wide Web+") OR (MH "Web Browsers") OR "web" | 136,894 | [Edit](javascript:__doPostBack('ctl00$ctl00$MainContentArea$MainContentArea$editControl$printHistory$HistoryRepeater$ctl21$linkEditSearch',''))S22 |
| S23 | S12 OR S13 OR S14 OR S15 OR S16 OR S17 OR S18 OR S19 OR S20 OR S21 OR S22 | 852,248 | [Edit](javascript:__doPostBack('ctl00$ctl00$MainContentArea$MainContentArea$editControl$printHistory$HistoryRepeater$ctl22$linkEditSearch',''))S23 |
| S24 | S3 AND S11 AND S23 | 198 |  |
