# Supplementary material for: How effective are digital interventions in increasing flu vaccination among pregnant women? A systematic review and meta-analysis
Source: J Public Health (Oxf). 2021 Jun 23;44(4):863–76. doi: 10.1093/pubmed/fdab220 (PMC9715302; doi:10.1093/pubmed/fdab220)
Supplement: Supplemental_2_flowchart_fdab220 [file supplemental_2_flowchart_fdab220.docx]

Supplemental 2: PRISMA flow diagram

Records excluded
(n =487)

Records screened
(n = 521)

Full-text articles excluded, with reasons
(n =24)

Wrong population (n= 7)

Commentry/ review (4)

Conference presentation (n= 4)

Wrong intervention (n=3)

Wrong outcome (n= 3)

Wrong study design (2)

Full-text articles assessed for eligibility
(n = 34)

Records after duplicates removed
(n = 521)

Additional records identified through other sources
(n = 42)

## Identification

## Eligibility

## Included

## Screening

Records identified through database searching
(n =481)

Studies included in synthesis
(n =10)

Studies included in meta-analysis
(n = 10)
