# Supplementary figures and images for: How effective are digital interventions in increasing flu vaccination among pregnant women? A systematic review and meta-analysis
Source: J Public Health (Oxf). 2021 Jun 23;44(4):863–76. doi: 10.1093/pubmed/fdab220 (PMC9715302; doi:10.1093/pubmed/fdab220)

Supplemental 3:

Figure 1: Meta-analysis and forest plot


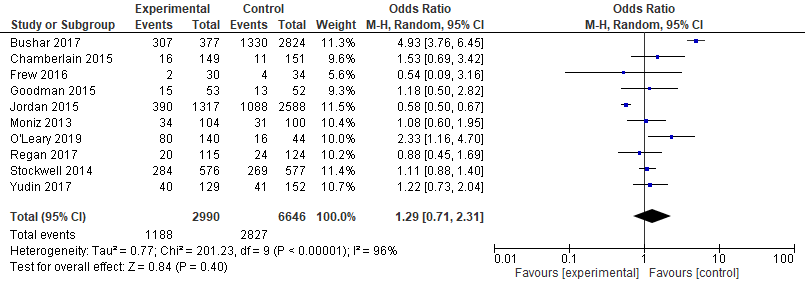

Supplement: Supplemental_3_forest_plots_fdab220 [file supplemental_3_forest_plots_fdab220.docx]

Supplemental 4: Forest plot of Sensitivity analysis: Removing high risk of bias studies


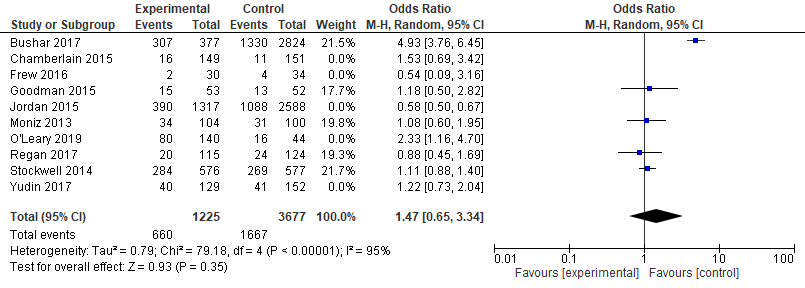

Supplement: Supplemental_4_sensitivity_forest_plot_fdab220 [file supplemental_4_sensitivity_forest_plot_fdab220.docx]

Supplemental 4: Funnel Plot


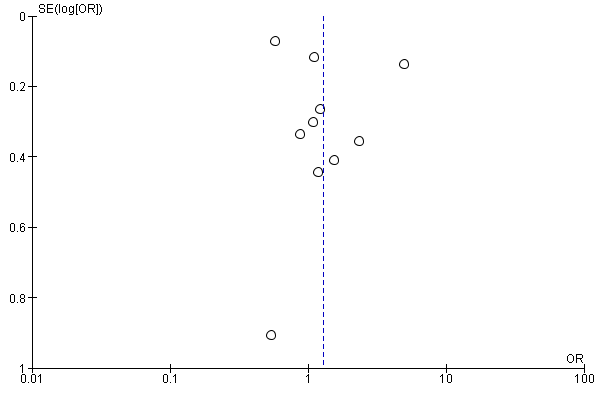

Supplement: Supplemental_6_funnel_plot_fdab220 [file supplemental_6_funnel_plot_fdab220.docx]
