# Supplementary material for: How effective are digital interventions in increasing flu vaccination among pregnant women? A systematic review and meta-analysis
Source: J Public Health (Oxf). 2021 Jun 23;44(4):863–76. doi: 10.1093/pubmed/fdab220 (PMC9715302; doi:10.1093/pubmed/fdab220)

Supplemental 5: Forest plot of Moderator analysis: Text message interventions


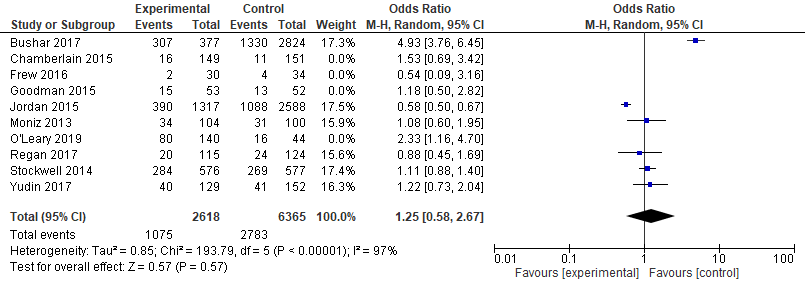


Other interventions


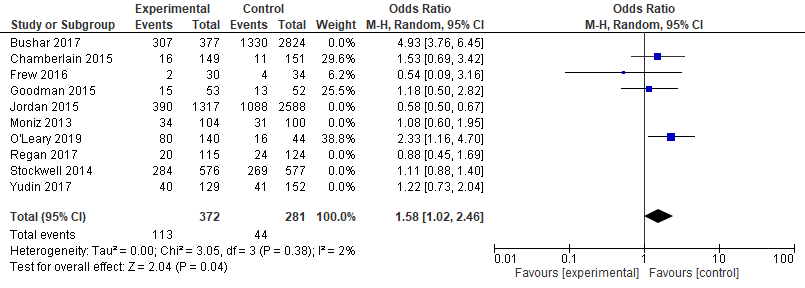

Supplement: Supplemental_5_moderator_analysis_forest_plot_fdab220 [file supplemental_5_moderator_analysis_forest_plot_fdab220.docx]
